# Prevalence and spectrum of *BRCA* germline variants in mainland Chinese familial breast and ovarian cancer patients

**DOI:** 10.18632/oncotarget.7144

**Published:** 2016-02-02

**Authors:** Yeong C. Kim, Linli Zhao, Hanwen Zhang, Ye Huang, Jian Cui, Fengxia Xiao, Bradley Downs, San Ming Wang

**Affiliations:** ^1^ Department of Genetics, Cell Biology and Anatomy, College of Medicine, University of Nebraska Medical Center, Omaha, NE, USA

**Keywords:** germline variant, BRCA1, BRCA2, mainland Chinese, familial breast and ovarian cancer

## Abstract

Germline mutations in *BRCA1* and *BRCA2* are the most penetrating genetic predispositions for breast and ovarian cancer, and their presence is largely ethnic-specific. Comprehensive information about the prevalence and spectrum of *BRCA* mutations has been collected in European and North American populations. However, similar information is lacking in other populations, including the mainland Chinese population despite its large size of 1.4 billion accounting for one fifth of the world's population. Herein, we performed an extensive literature analysis to collect *BRCA* variants identified from mainland Chinese familial breast and ovarian cancer patients. We observed 137 distinct *BRCA1* variants in 409 of 3,844 and 80 distinct *BRCA2* variants in 157 of 3,024 mainland Chinese patients, with an estimated prevalence of 10.6% for *BRCA1* and 5.2% for *BRCA2*. Of these variants, only 40.3% in *BRCA1* and 42.5% in *BRCA2* are listed in current Breast Cancer Information Core database. We observed higher frequent variation in *BRCA1* exons 11A, 11C, 11D, and 24 and *BRCA2* exon 10 in Chinese patients than in the patients of other populations. The most common pathogenic variant in *BRCA1* wasc.981_982delAT in exon 11A, and in *BRCA2* c.3195_3198delTAAT in exon 11B and c.5576_5579delTTAA in exon 11E; the most common novel variant in *BRCA1* was c.919A>G in exon 10A, and in *BRCA2* c.7142delC in exon 14. None of the variants overlap with the founder mutations in other populations. Our analysis indicates that the prevalence of *BRCA* variation in mainland Chinese familial breast and ovarian cancer patients is at a level similar to but the spectrum is substantially different from the ones of other populations.

## INTRODUCTION

*BRCA1* and *BRCA2 (BRCA)* are rapidly evolving genes with high levels of variation across primate species [1-3]. Germline mutations in *BRCA* predispose individuals for breast and ovarian cancer [4-5]. Extensive efforts have been made to determine the prevalence and spectrum of germline mutations in both genes to aid clinical diagnosis of and prevent the disease [6-8].

Increasing evidence indicates that the presence of *BRCA* germline mutations in human familial breast and ovarian cancer is largely ethnic-specific [9]. For example, 185delAG [c.66_67delAG according to human genome variation society (HGVS) nomenclature] and 5382insC (c.5263_5264insC) in *BRCA1* and 6174delT (c.5946delT) in *BRCA2* are highly prevalent in Ashkenazi Jews [10]; c.4153delA (c.4035delA), C61G (c.-58C>G), and 5382insC (c.5263_5264insC) in *BRCA1* are common in Polish familial breast cancer patients [11]; c.303T>G, c.5324T>G, c.1623dupG, and c.4122_4123delTG in *BRCA1* are frequently present in the familial breast cancer patients of African ancestry [12]; ex9-12del in *BRCA1* is often seen in Mexican familial breast and ovarian cancer patients [13], and c.7480C>T in *BRCA2* is enriched in Korean familial breast cancer patients [14].

*BRCA* mutations have been extensively analyzed in European and North American populations, but much less are known about them in Asian, African, and Latin American populations, although these contribute most of the total human population. Using the data from Western populations to interpret *BRCA* mutations in non-Western patients can be inaccurate and lead to misdiagnoses. Therefore, knowledge of ethnic-specific *BRCA* mutations is urgently demanding and will be highly beneficial for the patients.

**Figure 1:**
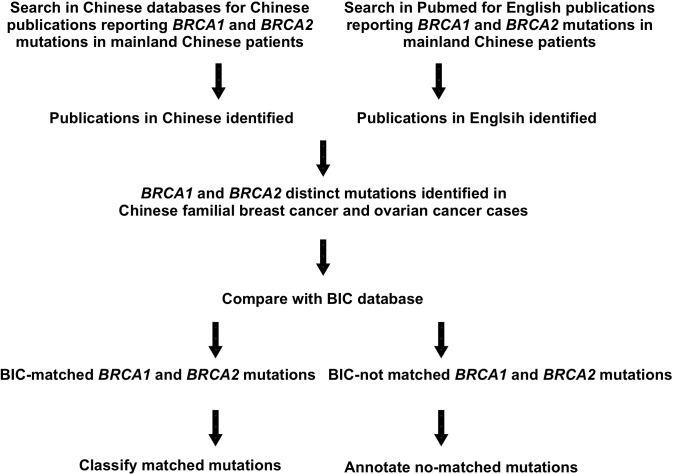
Outline of the study It shows the steps taken to extract information about *BRCA* variation in mainland Chinese familial breast and ovarian cancer patients.

Mainland China has a population size of nearly 1.4 billion, accounting for one fifth of the human population worldwide. However, limited information about *BRCA* mutations in this large population is available in current *BRCA* variation databases. For example, only 13 of the 1,791 *BRCA1* variants and three of the 2,000 *BRCA2* variants in the Breast Cancer Information Core (BIC) database were derived exclusively from mainland Chinese patients [15]. We hypothesized that 1) *BRCA* variation may be common in this population, and 2) many variants representing potential mutations may have already been identified but this information is unknown outside the Chinese scientific community, because many Chinese scientists publish in Chinese rather than in English and most Chinese medical and health science journals are not included in international journal databases [16]. To test our hypothesis, we performed an extensive survey of Chinese and English scientific literature to collect *BRCA* variant data derived solely from mainland Chinese familial breast and ovarian cancer patients (Figure 1).

## RESULTS AND DISCUSSION

### Identification of publications

We identified 32 Chinese publications, including 24 peer-reviewed papers and eight graduate theses (Supplementary Table 1), and 11 peer-reviewed English papers. This totaled 43 publications covering between 2003 and 2015 reported *BRCA* variants from mainland Chinese familial breast and ovarian cancer patients [17-59].

From these publications, we identified familial breast cancer cases using the inclusion criteria described in each publication: at least one first-degree relative with breast cancer irrespective of age; breast cancer diagnosed before the age of 35 years with a family history of breast and/or ovarian cancer; at least one or two first- or second-degree relatives diagnosed with breast cancer at any age; at least three relatives affected by breast cancer or breast and ovarian cancer; triple-negative breast cancer patients diagnosed before the age of 45 years; bilateral breast cancer diagnosed before the age of 50 years; one or more primary breast/ovarian cancers in first- or second-degree relatives; and at least one relative with cancer other than breast and ovarian cancer that is known to be *BRCA1*-related. From these publications, we also collected pedigree and genotype information available from family members although most publications only analyzed the proband without such information (Supplementary Table 2).

**Figure 2:**
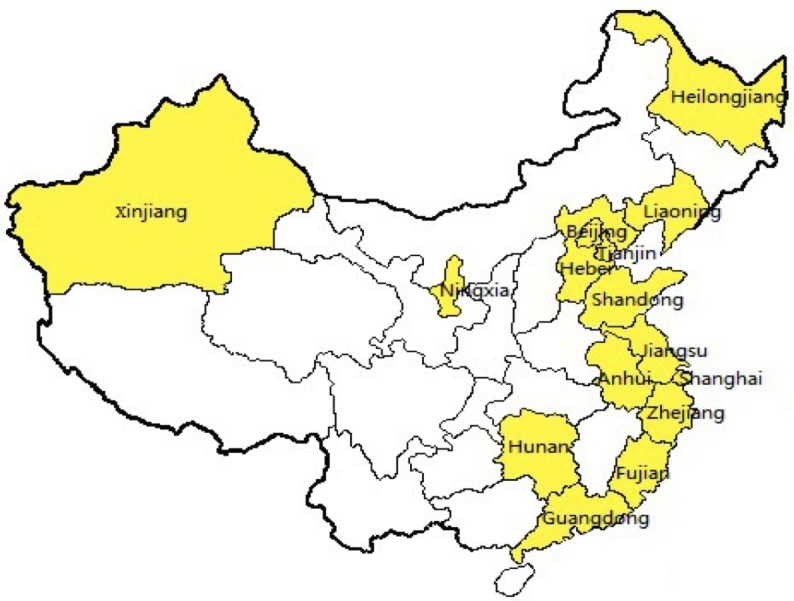
Geographic locations of the original studies The original studies were performed in 15 provinces and cities in mainland China. Of these, 13 were in east coast area of Han Chinese and two were in Xinjiang and Ningxia of other ethnic groups.

We identified a total of 3,844 familial breast and ovarian cancer cases from the original studies. All of these were analyzed for *BRCA1* (3,129 covered all exons), and 3,024 were analyzed for *BRCA2* (2,854 covered all exons); 92% of the 3,844 cases were Han Chinese and the rest were from other ethnic groups (Hui, Mongol, Uyghur, Kazakh, and Russian) (Table 1). These studies were performed in 15 provinces or cities in mainland China, mostly in the densely populated, economically advanced eastern coast area, with the exception of Xinjiang and Ningxia regions (Figure 2). The information highlights the need to analyze the population in so far uncovered regions to fully determine the prevalence and spectrum of *BRCA* mutations in the entire mainland Chinese population.

**Table 1:** Publications reporting BRCA mutations in mainland Chinese patients

Year	Location	Ethnicity	Cases	Targeted exons		Published in		Methods*	References
				BRCA1	BRCA2	Chinese	English		
2003	Beijing	Han	9	All		+		a	17
2003	Beijing	Han	26	All			+	a	18
2003	Jiangsu	Han	23	All but 1, 4		+		a	19
2003	Shanghai	Han	20	All			+	a	20
2003	Beijing	Han	15	4, 8, 11, 18, 19, 20	All but 15, 16, 25, 26	+		b	21
2004	Shanghai	Han	645	All	All		+	b	22
2005	Shanghai	Han	13	All	All	+		a	23
2005	Anhui	Han	76	All but 1, 3, 4, 6, 7, 10, 14, 19, 21-24		+		a	24
2006	Shanghai	Han	35	All	All	+		a	25
2006	Shanghai	Han	33	All	All		+	a	26
2007	Hebei	Han	18	2, 11A, 11B, 20	Not specified 4 mutations	+		c	27
2007	Shanghai Liaoning Shadong	Han	177	Not specified 7 mutations		+		a	28
2007	Shanghai Liaoning Shadong	Han	39	All		+		a	29
2007	Shanghai Liaoning Shadong	Han	60	1100delAT, IVS17-1G>T, IVS21+1G>C, 5640delA		+		a	30
2007	Shanghai Liaoning Shadong	Han	139	All	All	+		a	31
2007	Guangdong	Han	17	All		+		a	32
2008	Shanghai Liaoning Shandong	Han	115	All	All	+		a	33
2008	Shanghai Liaoning Shadong	Han	489	All	All		+	d	34
2008	Shandong	Han	25	All	All	+		a	35
2008	Shanghai Guangdong Liaoning	Han	219	All	All	+		a	36
2009	Shandong	Han	25	All		+		a	37
2009	Beijing	Han	139	All			+	a	38
2009	Hunan	Han	26	All	All	+		a	39
2009	Fujian	Han	20	11		+		b	40
2009	Tianjin	Han	5	1, 11, 16, 20		+		a	41
2009	Shandong	Han	30	2, 20		+		a	42
2010	Heilongjiang	Han	54	All but 1, 4		+		c	43
2011	Shandong	Han	8	2, 11		+		b	44
2012	Hebei	Han	13	2, 11, 20	11	+		c	45
2012	Hebei	Han	64	All	All	+		a	46
2012	Ningxia	Hui	7	5, 11, 18, 20, 24	10, 11	+		b	47
2012	Guangdong	Han	92	All but 1, 4		+		b	48
2012	Beijing	Han	409	All	All		+	b	49
2012	Zhejiang	Han	92	3, 8, 11, 12, 13, 24	3, 5, 6, 10, 11, 18, 22, 23	+		b	50
2013	Zhejiang	Han	62	All	All	+		b	51
2013	Xinjiang	Han	30	All	All	+		a	52
2013	Xinjiang	Han	79	All	All		+	a	53
2014	Xinjiang	Han Mongol Hui Uygur	214	All	All	+		a	54
2014	Xinjiang	Han	25	Not specified	Not specified		+	a	55
2014	Shanghai	Han	2	All	All	+		e	56
2015	Xinjiang	Han Mongol Hui Uygur Kazakh Russian	82	All	All	+		a	57
2015	Beijing	Han	109	All	All		+	b	58
2015	Shanghai	Han	64	All	All		+	e	59
Total			3,844	3844	3024	32	11		43

* a. DHPLC, Sanger sequencing; b. Sanger sequencing; c. SSCP, Sanger sequencing; d. SSCP, DHPLC, Sanger sequencing; e. NGS, Sanger sequencing

Multiple assays including hetero-duplex formation, single-strand conformation polymorphism (SSCP), denaturing high-performance liquid chromatography (DHPLC), and Sanger sequencing were used in the original studies. All *BRCA1* and *BRCA2* variants collected in our current study were identified by either direct Sanger sequencing or by Sanger sequencing validation for the results from other assays (Table 1).

### *BRCA* variants identified from publications

By mining the variant data from the 3,844 cases, we identified a total of 137 distinct *BRCA1* variants in 409 cases, and 80 distinct *BRCA2* variants in 157 cases (Table 2, Supplementary Table 3; Table 3, Supplementary Table 4). Of the 137 *BRCA1* variants, 33 (24.6%) were detected by at least two different studies; of the 80 *BRCA2* variants, 22 (27.2%) were detected by at least two different studies.

**Table 2:** Examples of *BRCA1* variants identified in mainland Chinese familial breast and ovarian cancer patients*

Class (BIC)	Exon	HGVS annotation		Variation type	Total case	Carrier
		cDNA	Protein			
Class 5	11A	c.981_982delAT	p.Cys328*	Frameshift	1142	18
Class 5	11A	c.1116G>A	p.Trp372*	Nonsense	480	5
Class 5	11B	c.2110_2111delAA	p.Asn704Cysfs*7	Frameshift	822	8
Class 5	11B	c.2275C>T	p.Gln759*	Nonsense	473	5
Class 5	11B	c.1556delA	p.Lys519Argfs*13	Frameshift	7	2
Class 5	11B	c.2138C>G	p.Ser713*	Nonsense	50	2
Class 5	11D	c.3531delT	p.Phe1177Leufs*33	Frameshift	7	6
Class 5	11D	c.3916_3917delTT	p.Leu1306Aspfs*23	Frameshift	518	3
Class 5	11D	c.3640G>T	p.Glu1214*	Nonsense	239	3
Class 5	11D	c.3607C>T	p.Arg1203*	Nonsense	239	2
Class 5	11D	c.4065_4068delTCAA	p.Asn1355Lysfs*10	Frameshift	171	2
Class 5	11D	c.3770_3771delAG	p.Glu1257Glyfs*9	Frameshift	548	2
Class 5	19	c.5154G>A	p.Trp1718*	Nonsense	62	2
Pending	I-5	c.212+1G>T	-	IVS	214	3
Pending	11A	c.1064A>G	p.Lys355Arg	Missense	92	8
Pending	11B	c.2077G>A	p.Asp693Asn	Missense	214	3
Pending	11B	c.1934C>A	p.Ser645Tyr	Missense	214	2
Pending	11C	c.3113A>G	p.Glu1038Gly	Missense	437	31
Pending	11C	c.3119G>A	p.Ser1040Asn	Missense	667	3
Pending	11D	c.3548A>G	p.Lys1183Arg	Missense	439	34
Pending	11D	c.3508A>T	p.Ile1170Phe	Missense	76	2
Pending	I-16	c.4986+1G>A	-	IVS	101	2
Pending	16	c.4837A>G	p.Ser1613Gly	Missense	302	17
Pending	22	c.5363G>T	p.Gly1788Val	Missense	548	2
Pending	24	c.5470_5477delATTGGGCA	p.Ile1824Aspfs*3	Frameshift	1505	20
Pending	24	c.5521delA	p.Ser1841Valfs*2	Frameshift	1272	8
Pending	24	c.5503C>T	p.Arg1835*	Nonsense	173	2
Novel	2	c.-1A>T	-	IVS	76	2
Novel	11A	c.919A>G	p.Lys307Glu	Missense	92	10
Novel	11A	c.1660G>T	p.Glu554*	Nonsense	628	3
Novel	11B	c.2073delA	p.Arg691Serfs*10	Frameshift	430	5
Novel	11B	c.2248_2252delCTCAT	p.Leu750Valfs*10	Frameshift	518	2
Novel	11C	c.2572C>T	p.Gln858*	Nonsense	782	4
Novel	11C	c.3122C>G	p.Ser1041*	Nonsense	743	4
Novel	11C	c.2798_2799delGT	p.Gly933Alafs*4	Missense	743	3
Novel	11C	c.3294delT	p.Pro1099Leufs*10	Frameshift	239	3
Novel	11C	c.2939T>A	p.Ile980Lys	Missense	214	2
Novel	11C	c.2941C>G	p.Pro981Ala	Missense	214	2
Novel	11C	c.2603C>A	p.Ser868*	Nonsense	480	2
Novel	11D	c.3363_3367delTACAG	p.Asn1121Lysfs*10	Frameshift	1186	7
Novel	11D	c.3359_3363delTTAAT	p.Val1120Aspfs*11	Frameshift	1226	5
Novel	11D	c.3432G>C	p.Gln1144His	Missense	7	3
Novel	11D	c.3450delT	p.Asp1151Metfs*4	Frameshift	519	3
Novel	11D	c.3952A>C	p.Ile1318Leu	Missense	20	2
Novel	11D	c.3433delG	p.Val1145Phefs*10	Frameshift	7	2
Novel	I-23	c.5468-1_5474delGCAATTGG	-	IVS	823	8

* The table lists the variants detected in at least two cases in each class

**Table 3:** Examples of *BRCA2* variants identified in mainland Chinese familial breast and ovarian cancer patients*

Class (BIC)	Exon	HGVS annotation		Variant type	Total cases	Carrier
		cDNA	Protein			
Class 5	3	c.262_263delCT	p.Leu88Alafs*12	Frameshift	518	2
Class 5	10	c.1832C>A	p.Ser611*	Nonsense	518	4
Class 5	10	c.1399A>T	p.Lys467*	Nonsense	191	3
Class 5	19	c.8485C>T	p.Gln2829*	Nonsense	99	2
Class 5	11B	c.3195_3198delTAAT	p.Asn1066Leufs*10	Frameshift	1226	5
Class 5	11B	c.2808_2811delACAA	p.Ala938Profs*21	Frameshift	708	2
Class 5	11C	c.3744_3747delTGAG	p.Ser1248Argfs*10	Frameshift	708	2
Class 5	11D	c.5164_5165delAG	p.Ser1722Tyrfs*4	Frameshift	518	4
Class 5	11E	c.5576_5579delTTAA	p.Ile1859Lysfs*3	Frameshift	1302	5
Class 5	11E	c.5682C>G	p.Tyr1894*	Nonsense	99	2
Class 5	11F	c.6591_6592delTG	p.Glu2198Asnfs*4	Frameshift	409	3
Class 5	23	c.9098_9099insA	p.Gln3034Serfs*10	Frameshift	518	4
Pending	10	c.865A>C	p.Asn289His	Missense	321	13
Novel	10	c.1303dupA	p.Arg435Lysfs*17	Frameshift	708	2
Novel	10	c.1881delA	p.Pro628Hisfs*16	Frameshift	708	2
Novel	11A	c.2442delC	p.Met815Trpfs*10	Frameshift	708	2
Novel	11E	c.5864C>G	p.Ser1955*	Nonsense	409	2
Novel	11F	c.6645_6648CTCC	p.Tyr2215*	Nonsense	375	3
Novel	11F	c.6150_6151insT	p.Asn2051*	Nonsense	109	2
Novel	14	c.7142delC	p.Pro2381Hisfs*13	Frameshift	99	5
Novel	18	c.8172delG	p.Trp2725Glyfs*8	Frameshift	109	3
Novel	18	c.8234dupT	p.Thr2746Aspfs*18	Frameshift	708	2
Novel	19	c.8400_8403del4ins5	p.Phe2801Leufs*10	Nonsense	409	2
Novel	20	c.8517C>A	p.Tyr2839*	Nonsense	181	2
Novel	22	c.8820_8823del	p.Gln2941Leufs*34	Frameshift	1013	3
Novel	22	c.8950delT	p.Ser2984Glnfs*4	Frameshift	604	2
Novel	23	c.9105dup	p.Gln3036Serfs*8	Frameshift	109	4
Novel	24	c.9253delA	p.Thr3085Glnfs*19	Frameshift	375	3

* The table lists the variants detected in at least two cases in each class

### Prevalence assessment

The prevalence of the variant carriers was 10.6% (409/3,844) for *BRCA1* and 5.2% (157/3,024) for *BRCA2* (Of the 3,844 cases, all were used for *BRCA1*, but 3,024 were used for *BRCA2*). The total number of cases used for all exon analysis was 3,129 in *BRCA1* and 2,854 in *BRCA2*. Thus, the total number of cases in the *BRCA2* group accounted for 91.2% of the *BRCA1* (2,854/3,129). Therefore, the different prevalence of *BRCA1* and *BRCA2* variations is unlikely caused by the analysis of different cases in each group but instead reflects the fact that *BRCA1* has a higher prevalence than *BRCA2* in Chinese population. This pattern differs from that in the neighboring Korean population, which has a much higher prevalence of *BRCA2* variation than *BRCA1* variation [14]. The variation types included frameshift, nonsense, missense, and splicing changes. Majority of the variants except a few do not have frequency information in genome databases, indicating that the variants are mostly rare in human population (Supplementary Table 3, Supplementary Table 4).

### Exon distribution of *BRCA* variants between Chinese and other patient populations

**Figure 3:**
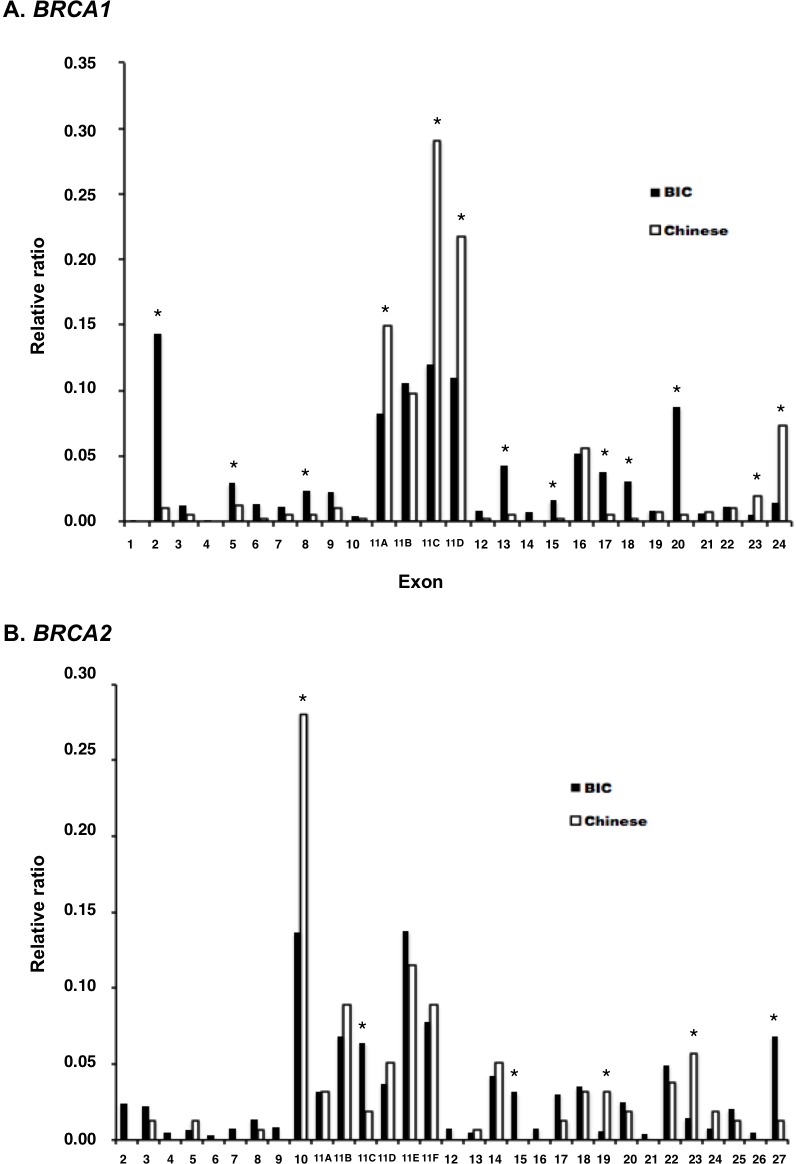
Comparison of exon distribution frequencies of *BRCA* variation between mainland Chinese and BIC populations Relative ratios between these two datasets were used for the comparison (see text for the details). Chi square (*χ*^2^) and Fisher exact test were used for statistics analysis. “*” refers to *p* < 0.05 (actual P values listed in Supplementary Table 5). **A.** Variant distribution in *BRCA1*. **B.** Variant distribution in *BRCA2*.

We compared exon distribution frequencies of *BRCA* variations between mainland Chinese patients and other patient populations represented in the BIC database. We compared the ratios calculated as: number of variation cases in each exon / total number of variation cases in each data set. The total number of variation cases (entries) in the BIC dataset was 15,311 for *BRCA1* [61] and 14,914 for *BRCA2* [62]; the total number of variation cases in this study was 409 for *BRCA1* and 157 for *BRCA2*. The results showed that the distribution frequencies in 13 out of 24 *BRCA1* exons were significantly different between between mainland Chinese and BIC populations (Figure 3A). Variants in mainland Chinese were particularly lower in exons 2 and 20 but higher in exons 11A, 11C, and 11D (exon 11 is arbitrarily divided into 11A, 11B, 11C, and 11D by the BIC database because of its large size) and exon 24 than in other populations. The variants in *BRCA1* exons 11A, 11C, 11D and exon 24 occurred in 299 of the 409 (73.1%) Chinese *BRCA1-* variation cases. In *BRCA2*, the differences were smaller with only 6 out of 27 exons showed significant difference between mainland Chinese and BIC populations. Exon 10 was the highest in mainland Chinese with 44 of the 157 (28%) Chinese *BRCA2*-variation cases (Figure 3B). Therefore, *BRCA1* exon 11A, 11C, 11D, exon 24, and *BRCA2* exon 10 are the variation hot spots in mainland Chinese patients.

### BIC-matched variants

**Figure 4:**
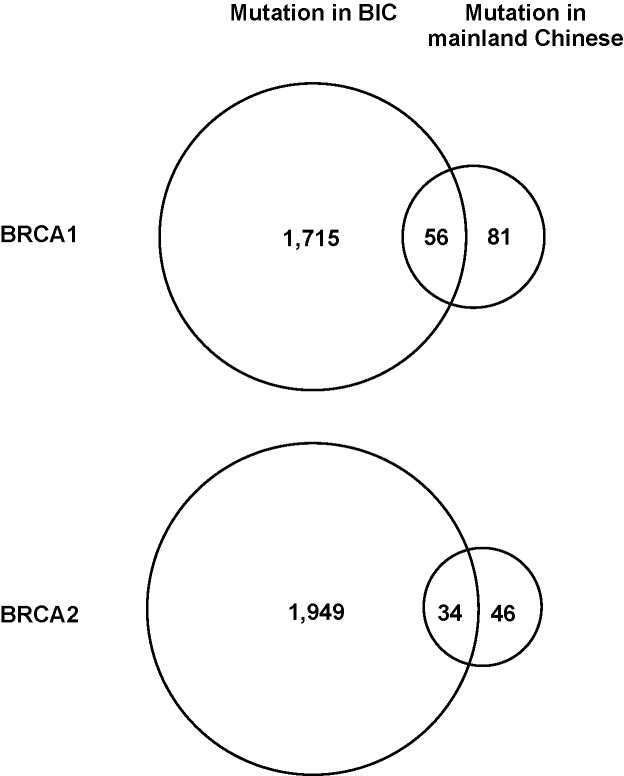
Matching *BRCA* variants to the BIC database The 137 *BRCA1* and 80 *BRCA2* distinct variants from mainland Chinese patients were compared with the 1,781 *BRCA1* and 2,000 *BRCA2* distinct variants in the BIC database. Of the Chinese variants, 56 *BRCA1* and 34 *BRCA2* variants were matched, whereas 82 *BRCA1* and 46 *BRCA2* variants were not.

Fifty-six (40.3%) *BRCA1* and 34 (42.5%) *BRCA2* variants exist in the BIC database (Figure 4). Of these, 27 *BRCA1* and 23 *BRCA2* variants are classified by BIC as Class 5 (Pathogenic), 27 *BRCA1* and 9 *BRCA2* variants as Pending [most were variants of unknown significance (VUS)], and two *BRCA1* and two *BRCA2* variants as Class 1 (Benign). The most common pathogenic *BRCA1* variant was c.981_982delAT (p.Cys328*) in exon 11A (*n* = 18), confirming the previous observation in a smaller group of patients [30]. The frequency of this variant was substantially higher in mainland Chinese than in non-Chinese populations: 18 of 409 (4.4%) Chinese *BRCA1* variant carriers carried this variant, compared with only 18 of 15,311 (0.1%) *BRCA1* variant carriers in the BIC database. The most common *BRCA1* Pending variant was c.3548A>G (p.Lys1183Arg) in exon 11D (*n* = 34; frequency in 1000 Genomes: 0.3526) and c.3113A>G (p.Glu1038Gly) in exon 11C (*n* = 31, frequency in 1000 Genomes: 0.3357, in Han Chinese Beijing: 0.689). Three Pending variants [c.5470_5477delATTGGGCA (p.Ile1824Aspfs*3), c.5503C>T (p.Arg1835*)] c.5521delA (p.Ser1841Valfs*2) with high frequencies were located at exon 24, which contributes to the BRCT domain of BRCA1 (Figure 3A). The most common Pathogenic *BRCA2* variant was c.3195_3198delTAAT (p.Asn1066Leufs*10) in exon 11B (*n* = 5) and c.5576_5579delTTAA (p.Ile1859Lysfs*3) in exon 11E (*n* = 5), and the most common Pending variant was c.865A>C (p.Asn289His) in exon 10 (*n* = 13; frequency in 1000 Genomes: 0.0737). Except for the *BRCA1* c.981_982delAT variant, other known pathogenic and Pending variants in either *BRCA1* or *BRCA2* are unlikely to be founder mutation candidates among mainland Chinese patients due to their lower prevalence or higher frequency in normal population.

No variants were found to overlap with other ethnic-specific *BRCA* founder mutations, including *BRCA1* 185delAG (c.66_67delAG, HGVS nomenclature) and 5382insC (c.5263_5264insC) and *BRCA2* 6174delT (c.5946delT) in Ashkenazi Jews [10]; *BRCA1* c.4153delA (c.4035delA), C61G (c.-58C>G), and 5382insC (c.5263_5264insC) in Poles [11]; *BRCA1* c.303T>G, c.5324T>G, c.1623dupG, and c.4122_4123delTG in Africans [12]; *BRCA1* ex9-12del in Mexicans [13], and BRCA2 c.7480C>T in Koreans [14].

### Novel *BRCA* variants

Eighty-one (59.4%) *BRCA1* variants and 46 (57.5%) *BRCA2* variants are not recorded in the BIC database (Figure 4). A total of 19 of these 81 *BRCA1* variants and 15 of the 46 *BRCA2* variants were detected in at least two cases, with c.919A>G (p.Lys307Glu) in *BRCA1* exon 11A (*n* = 10), c.7142delC (p.Pro2381Hisfs*13) in *BRCA2* exon 14 (*n* = 5) having the highest frequencies. We compared these novel variants with the *BRCA* variant dataset from Asian populations [60], and identified 35 overlapping variants (18 in *BRCA1* and 17 in *BRCA2*). Thirty-three (94.3%) of these overlapping variants were from Chinese ethnicity but not from other ethnicities (Supplementary Table 6), confirming that these novel variants are mainland Chinese-specific. The presence of multiple novel variants provides a rich resource to identify new *BRCA* pathogenic mutations in mainland Chinese population.

In conclusion, our study indicates that *BRCA* variations are common in mainland Chinese familial breast and ovarian cancer patients. The absence of such information in current international BRCA databases appears to largely reflect the poor communication between Western and Chinese scientific communities. Our study also indicates while the prevalence of *BRCA* variation is similar to that of other populations, the spectrum of *BRCA* variation in Chinese patients differs substantially with the hot spots of *BRCA1* exons 11A, 11C, 11D, 24 and *BRCA2* exon 10. Except the c.981_982delAT in *BRCA1* exon 11A, there is no strong evidence showing the presence of common founder *BRCA* mutations in mainland Chinese patients, although such a possibility may exist in certain subpopulations of specific geographic regions or ethnic groups in mainland China.

## MATERIALS AND METHODS

### Information sources

We searched two major Chinese scientific databases, China National Knowledge Infrastructure (CNKI) [63] and WanFang [16], which comprehensively collect information from Chinese academic journals, dissertations, conference proceedings, and patents, by using the key words “breast cancer”, “*BRCA1* mutation”, and “*BRCA2* mutation” in Chinese characters. From the identified publications, we excluded those of sporadic breast cancer, animals, and those about patients marked with “early diagnosis”, “triple-negative”, and “bilateral” but without age indication, “male”, and from non-mainland Chinese. Using similar approaches but in English, we also searched the PubMed database to identify non-Chinese publications reporting *BRCA* mutations from mainland Chinese patients (Figure 1).

We applied multiple steps to ensure the reliability of the identified variants, including: 1) only including variants detected or validated by Sanger sequencing; 2) re-annotating all variants following HGVS nomenclature using the reference sequences U14680 for *BRCA1* and U43746 for *BRCA2*, regardless of original annotation; 3) using the BIC database (13-Mar-2015 version) as a reference to classify variants as known variants with BIC designation or novel variants without BIC designation; 4) excluding synonymous variants and un-interpretable variants from analysis; and 5) annotating novel variants by referring to their effects on coding changes in *BRCA1* and *BRCA2*. We used U14680 and U43746 as the reference sequences for *BRCA1* and *BRCA2* annotation, as they were used as the standard references by most of the cited publications and BIC database. However, different *BRCA* databases may use different *BRCA* reference sequences, which can generate differences for certain variants. For example, Clinvar database uses NM_007294 and NM_000059 as the references for *BRCA1 and BRCA2* (64). To facilitate data comparison with *BRCA* variants annotated by Clinvar database, we also included the variants annotated by using these two references (Supplementary Tables 3, 4). All variants were annotated following HGVS nomenclature.

## SUPPLEMENTARY TABLES







## Abbreviations

BIC: Breast Cancer Information Core
BRCA1: breast cancer 1, early onset
BRCA2: breast cancer 2, early onset
HGVS: human genome variation society
