# Supplementary material for: Prevalence and spectrum of BRCA germline variants in mainland Chinese familial breast and ovarian cancer patients
Source: Oncotarget. 2016 Feb 2;7(8):9600–12. doi: 10.18632/oncotarget.7144 (PMC4891063; doi:10.18632/oncotarget.7144)
Supplement: Supplementary file 1 [file oncotarget-07-9600-s001.pdf]

Supplementary table 1: Publications in Chinese

(The number refers to the reference number in the text)

17. 邓珊明,王怡,柯杨,徐光炜..9 例家族性乳腺癌患者 BRCA1 基因序列突变分析[J].北京大学学报(医学版),2003,35(4):373-376.DOI:10.3321/j.issn:1671-167X.2003.04.008.

19. 甄林林,武正炎,范萍等.家族性乳腺癌乳腺癌易感基因-1 突变分析[J].中华实验外科杂志,2003,20(2):170-171.DOI:10.3760/j.issn:1001-9030.2003.02.033.

21. 周远征,孙强,林守清,汪健,刘斌,李京湘等.中国汉族乳腺癌家系中 BRCA1 和 BRCA2 基因的胚系突变[J].中华医学杂志,2004,84(4):294-298.DOI:10.3760/j:issn:0376-2491.2004.04.009.

23. 宋传贵,胡震,袁文涛,狄根红,沈镇宙,黄薇等.上海地区早发性乳腺癌患者 BRCA1 和 BRCA2 基因突变分析[J].中华医学杂志,2005,85(43):3030-3034.DOI:10.3760/j:issn:0376-2491.2005.43.003.

24. 吴永和.家族性乳腺癌易感基因（BRCA1）突变筛选及与病理表型关系的初步研究[D].中国协和医科大学,2005. (http://d.g.wanfangdata.com.cn/Thesis_Y761953.aspx)

25. 宋传贵,胡震,袁文涛,狄根红,沈镇宙,黄薇等.中国上海家族性乳腺癌 BRCA1 和 BRCA2 基因的突变[J].中华医学遗传学杂志,2006,23(1):27-31.DOI:10.3760/j.issn:1003-9406.2006.01.007.

27. 陈丽芬,耿翠芝,王桂兰,李军改等.河北省家族性和散发性乳腺癌易感基因 1/2 突变的研究[J].中华乳腺病杂志（电子版）,2010,04(4):418-426.DOI:10.3969/j.issn.1674-0807.2010.04.010.

28. 胡震,李文凤,柳晓义,张斌,曹明智,王永胜等.中国乳腺癌患者 BRCA1 基因的频发突变 5589del8[J].中华医学遗传学杂志,2007,24(4):378-381.DOI:10.3760/j.issn:1003-9406.2007.04.004.

29. 李文凤,胡震,柳晓义,张斌,曹明智,王永胜等.中国早发性乳腺癌患者中 BRCA1 基因突变分析[J].中华医学遗传学杂志,2007,24(5):499-504.DOI:10.3760/j.issn:1003-9406.2007.05.003.

30. 李文凤,胡震,柳晓义,张斌,曹明智,王永胜等.中国家族性乳腺癌人群中发现重复出现的 BRCA1 1100delAT 突变[J].中华医学杂志,2007,87(2):76-80.DOI:10.3760/j:issn:0376-2491.2007.02.002.

31. 李文凤.中国家族性/早发性乳腺癌人群中 BRCA1 和 BRCA2 基因突变的研究[D].复旦大学,2007.DOI:10.7666/d.y1171324.

32. 周婕,饶南燕,李顺荣,金亮,贾卫娟,龚畅,于风燕,苏逢锡,宋尔卫,邵志敏. 广东省家族性和早发性乳腺癌 BRCA1 基因突变的相关研究[J]. 南方医科大学学报,2009,02:213-216.

33. 胡震,李文凤,柳晓义,张斌,曹明智,王永胜等.家族性乳腺癌患者 115 例的 BRCA1 和 BRCA2 基因突变检测[J].中华医学杂志,2008,88(34):2383-2386.DOI:10.3321/j.issn:0376-2491.2008.34.003.
35. 马中良.山东半岛家族性和早发性乳腺癌 BRCA1/2 基因突变的研究[D].青岛大学,2008.DOI:10.7666/d.y1335403.
36. 饶南燕,周婕,赵林,胡震,狄根红,苏逢锡等. 219 例中国汉族遗传性乳腺癌患者 BRCA1 和 BRCA2 突变的研究[J].中国癌症杂志,2008,18(5):370-375.DOI:10.3969/j.issn.1007-3639.2008.05.010.
37. 曹明智,马中良,柳晓义,孔滨,李文凤,邵志敏等.山东东部地区家族性和早发性乳腺癌 BRCA1 基因突变的研究[J].现代生物医学进展,2009,9(6):1028-1030.
39. 黄隽,唐利立,胡震,夏婷,饶南燕,邵志敏等.中国湖南家族性和早发性乳腺癌 BRCA1 和 BRCA2 基因突变分析[J].中国癌症杂志,2008,18(8):566-572.DOI:10.3969/j.issn.1007-3639.2008.08.002.
40. 江雨,王文博,郭奇伟,葛运生,周裕林. 中国福建遗传性乳腺癌 BRCA1 基因突变分析[J]. 中国优生与遗传杂志,2009,07:20-22.
41. 孟洁,史玉荣,牛瑞芳,付丽. BRCAl 基因突变与年轻患者乳腺癌发生的相关性分析[J].中华医学杂志,2009,89(2):79-82.DOI:10.3760/cma.j.issn.0376-2491.2009.02.003.
42. 张磊.青岛地区家族性及早发性乳腺癌 BRCA1 基因第 2，20 外显子突变研究[D].青岛大学,2009.DOI:10.7666/d.y1469508.
43. 薛伟男,王劲松,宋燕妮,何川,张有学,庞达等. 家族性和早发性乳腺癌 BRCA1 基因突变分析[J].实用肿瘤学杂志,2010,24(5):408-412.DOI:10.3969/j.issn.1002-3070.2010.05.003.
44. 孔灵玲,崔文,王旭,姜小刚,张国安等.家族性乳腺癌患者 BRCA1 基因序列突变分析[J].济宁医学院学报,2011,34(2):96-98.DOI:10.3969/j.issn.1000-9760.2011.02.007.
45. 李军改,回天立,李峥,白杨,马国明,孙玉巧等.家族性乳腺癌家系成员乳腺癌易感基因突变的研究[J].中华乳腺病杂志（电子版）,2012,6(6):631-639.DOI:10.3877/cma.j.issn.1674-0807.2012.06.005.
46. 李峥.河北省家族性乳腺癌 BRCA1、BRCA2 基因突变相关研究[D].河北医科大学,2012.DOI:10.7666/d.y2105804.
47. 尹琳琳,陈耀平,杨宝珍,吴若芬,康毓芝. 回族家族性乳腺癌患者 BRCA1/BRCA2 基因序列突变分析[J]. 现代免疫学,2012,02:115-119.

48. 张文夏,王恩礼,谢建生,钟春娥,周颉,刘洁玲等.家族性和散发性乳腺癌患者 BRCA1 基因突变比较[J].中华临床医师杂志（电子版）,2012,(17):5305-5308.DOI:10.3877/cma.j.issn.1674-0785.2012.17.078.

50. 倪姗姗.浙江地区女性乳腺癌危险因素及 BRCA1/2 基因突变的研究[D].浙江大学,2012.(http://d.g.wanfangdata.com.cn/Thesis_Y2119858.aspx)

51. 曹文明. 中国乳腺癌高危人群 BRCA1/BRCA2 基因的胚系突变研究[D].浙江大学,2013.(http://d.g.wanfangdata.com.cn/Thesis_Y2385781.aspx)

52. 吴涛,王秀梅,欧江华,朱丽萍,许文婷,陈玲等.新疆维吾尔自治区高风险三阴性乳腺癌 30 例 BRCA 基因突变及临床病理特征对比[J].肿瘤研究与临床,2013,25(11):738-741.DOI:10.3760/cma.j.issn.1006-9801.2013.11.005.

54. 李涌涛. 新疆地区有遗传风险的乳腺癌患者相关易感基因 BRCA1/2、BRIP1、PALB2、CHEK2 的研究[D].新疆医科大学,2014.(http://www.cnki.net/KCMS/detail/detail.aspx?QueryID=0&CurRec=1&recid=&filename=1015544614.nh&dbname=CDFDLAST2015&dbcode=CDFD&pr=&urlid=&yx=&v=MTUxNDFYMUx1eFlTN0RoMVQzcVRyV00xRnJDVVJMK2ZiK2RtRkN2blc3ckJWRjI2RzdhOEd0Zk5xNUViUElSOGU=)

56. 吴霞,陈吉牟,狄文.应用第 2 代测序技术检测遗传性乳腺癌-卵巢上皮性癌综合征再发子宫浆液性腺癌患者的 BRCA1、BRCA2 基因突变[J].中华妇产科杂志,2014,(7):547-549.DOI:10.3760/cma.j.issn.0529-567x.2014.07.016.

57. 许文婷,伊丽娜,欧江华,吴涛.82 例新疆地区高风险遗传性乳腺癌患者 BRCA1/2 突变研究[J].医学信息,2015,(21):85-86.

Supplementary table 2: Pedigree and genotyping information

Number of families analysis	Genotypefamily members	Publication
9	Proband only	17
20	Proband only	20
645	Proband only	22
13	Proband only	23
76	Proband only	24
35	Proband only	25
139	Proband only	38
18	Proband only	27
177	Proband only	28
39	Proband only	29
139	Proband only	31
17	Proband only	32
489	Proband only	34
25	Proband only	35
219	Proband only	36
26	Proband only	39
20	Proband only	40
5	Proband only	41
30	Proband only	42
54	Proband only	43
8	Proband only	44
92	Proband only	48
409	Proband only	49
92	Proband only	50
62	Proband only	51
30	Proband only	52
79	Proband only	53
214	Proband only	54
25	Proband only	55
82	Proband only	57
109	Proband only	58
64	Proband only	59
60	Proband only, a BRCA1 variant confirmed to share with relatives in a single family	30
115	82, 25 and 8 cases from families with 2, 3, and 4 affected relatives. 8, 2 and 1 BRCA1 variants and 2 and 1 BRCA2 variants were shared	33
7	7 probands and 25 unaffected relatives. 7 BRCA1 variants confirmed to share in multiple families, 1 BRCA2 variant shared in all 7 families	47
64	64 probands with 34 unaffected relatives. One BRCA1 variant shared between proband and an unaffected family member	46
25	25 probands, 1 affected relative, and 10 unaffected relatives. 6 variants shared between affected and unafffected relatives	18
25	25 probands and 25 unaffected relatives	37

2	2 affected sisters and 1 unaffected daughter shared the same BRCA1 variant	56
33	18 probands with 2, 12 probands with 3, and 3 probands with 4 unaffected relatives, 4 BRCA1 and 1 BRCA2 variants were shared.	26
15	15 Probands and 8 unaffected relatives	19
14	14 probands. No shared variant identified	21
12	13 probands and 46 first-dgree unaffected relatives. No shared variants within families identified	45

Supplementary table 5: Exon distribution of *BRCA* variation between mainland Chinese and BIC

Exon	Cases		Ratio*		P value
	BIC	Chinese	BIC	Chinese	
BRCA1					
1	1	0	0.0001	0.0000	1.0000
2	2197	4	0.1435	0.0098	0.0000
3	187	2	0.0122	0.0049	0.2478
4	8	0	0.0005	0.0000	1.0000
5	445	5	0.0291	0.0122	0.0432
6	198	1	0.0129	0.0024	0.0607
7	164	2	0.0107	0.0049	0.3324
8	360	2	0.0235	0.0049	0.0130
9	349	4	0.0228	0.0098	0.0787
10	54	1	0.0035	0.0024	1.0000
11A	1252	61	0.0818	0.1490	0.0000
11B	1618	39	0.1057	0.0976	0.5975
11C	1835	119	0.1198	0.2900	0.0000
11D	1678	89	0.1096	0.2170	0.0000
12	118	1	0.0077	0.0024	0.3790
13	655	2	0.0428	0.0049	0.0002
14	111	0	0.0072	0.0000	0.1231
15	241	1	0.0157	0.0024	0.0309
16	790	23	0.0516	0.0561	0.6847
17	578	2	0.0378	0.0049	0.0005
18	466	1	0.0304	0.0024	0.0098
19	123	3	0.0080	0.0073	1.0000
20	1334	2	0.0871	0.0049	0.0000
21	89	3	0.0058	0.0073	0.5191
22	173	4	0.0113	0.0098	1.0000
23	74	8	0.0048	0.0195	0.0013
24	213	30	0.0139	0.0732	0.0000
Total	15311	409			
BRCA2					
2	355	0	0.0238	0.0000	0.0558
3	329	2	0.0221	0.0127	0.5891
4	73	0	0.0049	0.0000	1.0000
5	93	2	0.0062	0.0127	0.2604
6	48	0	0.0032	0.0000	1.0000
7	115	0	0.0077	0.0000	0.6358
8	200	1	0.0134	0.0064	0.7268
9	121	0	0.0081	0.0000	0.6402
10	2035	44	0.1364	0.2803	0.0000
11A	469	5	0.0314	0.0318	0.8134
11B	1012	14	0.0679	0.0892	0.2915
11C	955	3	0.0640	0.0191	0.0217
11D	556	8	0.0373	0.0510	0.3691
11E	2054	18	0.1377	0.1146	0.4036

11F	1155	14	0.0774	0.0892	0.5847
12	113	0	0.0076	0.0000	0.6346
13	73	1	0.0049	0.0064	0.5401
14	633	8	0.0424	0.0510	0.5991
15	468	0	0.0314	0.0000	0.0168
16	111	0	0.0074	0.0000	0.6334
17	453	2	0.0304	0.0127	0.3401
18	521	5	0.0349	0.0318	0.8340
19	89	5	0.0060	0.0318	0.0030
20	374	3	0.0251	0.0191	1.0000
21	61	0	0.0041	0.0000	1.0000
22	731	6	0.0490	0.0382	0.5326
23	210	9	0.0141	0.0573	0.0005
24	114	3	0.0076	0.0191	0.1230
25	302	2	0.0202	0.0127	0.7737
26	75	0	0.0050	0.0000	1.0000
27	1016	2	0.0681	0.0127	0.0059
Total	14914	157			

*ratio=number of variation cases in each exon / total number of variation cases in each data set

Supplementary table 6: Novel *BRCA* variants shared in Asian population

Exon	HGVS annotation		Total cases	Carrier	Reference [60]	
	cDNA	Protein			Location	Ethnicity
BRCA1						
8	c.463C>T	p.Gln155*	489	1	China	Chinese
11A	c.1279G>T	p.Glu427*	219	1	China	Chinese
11A	c.1660G>T	p.Glu554*	628	3	China	Chinese
11B	c.2010_2011insTG	p.Gly671Trpfs*31	1	1	China	Chinese
11B	c.2248_2252delCTCAT	p.Leu750Valfs*10	518	2	China	Chinese
11C	c.2572C>T	p.Gln858*	782	4	China	Chinese
11C	c.2603C>A	p.Ser868*	480	2	China	Chinese
11C	c.2671delT	p.Ser891Profs*2	1	1	China	Chinese
11C	c.2694dupA	p.Val899Serfs*4	1	1	China	Chinese
11C	c.2798_2799delGT	p.Gly933Alafs*4	743	3	China	Chinese
11C	c.3122C>G	p.Ser1041*	743	4	China	Chinese
11D	c.3359_3363delTTAAT	p.Val1120Aspfs*11	1217	6	China	Chinese
11D	c.3450delT	p.Asp1151Metfs*4	519	3	China	Chinese
I-17	c.5075-1G>T	-	489	1	China	Chinese
19	c.5161C>T	p.Gln1721*	62	1	Israel	Moslem
I-21	c.5332+1delG	-	1	1	China	Chinese
I-21	c.5332+1G>C	-	1	1	China	Chinese
I-23	c.5468-1_5474delGCAATTGG	-	823	8	China	Chinese
BRCA2						
5	c.464_468delGAGAT	p.Arg155Lysfs*26	41	1	China	Chinese
10	c.1082delA	p.Asn361Metfs*6	41	1	China	Chinese
10	c.1813delA	p.Ile605Tyrfs*9	708	2	China	Chinese
10	c.1881delA	p.Pro628Hisfs*16	725	3	China	Chinese
10	c.994_995dupA	p.Ile332Lysfs*18	17	1	China	Chinese
11A	c.1901delC	p.Ala634Valfs*18	219	1	China	Chinese
11A	c.2442delC	p.Met815Trpfs*10	772	4	China	Chinese
11D	c.5171delT	p.Ile1724Lysfs*17	409	1	China	Chinese
11E	c.5864C>G	p.Ser1955*	409	2	China	Chinese
11E	c.6033_6034delTT	p.Ser2012Glnfs*5	708	2	China	Chinese
11F	c.6800C>A	p.Ser2267*	409	1	China	Chinese
17	c.7963delC	p.Gln2655Asnfs*2	409	1	China	Chinese
19	c.8400_8403del4ins5	p.Phe2801Leufs*10	17	2	China	Chinese
22	c.8950delT	p.Ser2984Glnfs*4	743	3	China	Chinese
23	c.9007G>T	p.Gly3003*	409	1	China	Chinese
24	c.9253delA	p.Thr3085Glnfs*19	375	3	Korea	Korean
25	c.9356_9357delTAinsG	p.Leu3119*	17	1	China	Chinese
